# In-room computed tomography–based brachytherapy for uterine cervical cancer: results of a 5-year retrospective study

**DOI:** 10.1093/jrr/rrw121

**Published:** 2016-12-16

**Authors:** Tatsuya Ohno, Shin-Ei Noda, Noriyuki Okonogi, Kazutoshi Murata, Kei Shibuya, Hiroki Kiyohara, Tomoaki Tamaki, Ken Ando, Takahiro Oike, Yu Ohkubo, Masaru Wakatsuki, Jun-Ichi Saitoh, Takashi Nakano

**Affiliations:** 1 Department of Radiation Oncology, Gunma University Graduate School of Medicine, 3-39-22 Showa-machi, Maebashi, Gunma 371-8511, Japan; 2 Research Center for Charged Particle Therapy, National Institute of Radiological Sciences, 4-9-1 Anagawa, Chiba 263-8555, Japan

**Keywords:** cervical cancer, high-dose rate brachytherapy, image-based brachytherapy, in-room computed tomography, three-dimensional treatment planning

## Abstract

Herein, we investigate the long-term clinical outcomes for cervical cancer patients treated with in-room computed tomography–based brachytherapy. Eighty patients with Stage IB1–IVA cervical cancer, who had undergone treatment with combined 3D high-dose rate brachytherapy and conformal radiotherapy between October 2008 and May 2011, were retrospectively analyzed. External beam radiotherapy (50 Gy) with central shielding after 20–40 Gy was performed for each patient. Cisplatin-based chemotherapy was administered concurrently to advanced-stage patients aged ≤75 years. Brachytherapy was delivered in four fractions of 6 Gy per week. In-room computed tomography imaging with applicator insertion was performed for treatment planning. Information from physical examinations at diagnosis, and brachytherapy and magnetic resonance imaging at diagnosis and just before the first brachytherapy session, were referred to for contouring of the high-risk clinical target volume. The median follow-up duration was 60 months. The 5-year local control, pelvic progression-free survival and overall survival rates were 94%, 90% and 86%, respectively. No significant differences in 5-year local control rates were observed between Stage I, Stage II and Stage III–IVA patients. Conversely, a significant difference in the 5-year overall survival rate was observed between Stage II and III–IVA patients (97% *vs* 72%; *P* = 0.006). One patient developed Grade 3 late bladder toxicity. No other Grade 3 or higher late toxicities were reported in the rectum or bladder. In conclusion, excellent local control rates were achieved with minimal late toxicities in the rectum or bladder, irrespective of clinical stage.

## INTRODUCTION

The implementation of 3D image-guidance, treatment planning, and dose–volume histogram (DVH) parameter evaluation represents a major advancement in gynecological brachytherapy. Magnetic resonance imaging (MRI) is useful for treatment planning because it provides more accurate anatomical information than computed tomography (CT) [1]. Although MRI is the gold standard for 3D image-guided brachytherapy (IGBT) for cervical cancer, the transition to 3D treatment planning with MRI remains limited. Recent surveys on the use of IGBT for cervical cancer have revealed that CT and MRI are used for treatment planning in 15–65% and 1–21% of institutions in the American Brachytherapy Society, Canada, the UK, Australia, New Zealand and Japan [2].

An in-room CT on-rail brachytherapy system was installed at our institution in 2003. Since 2008, individualized 3D treatment planning of brachytherapy, supported by MRI at diagnosis and just before the first brachytherapy session, has been routinely used for patients with gynecological cancers. Herein, we investigate the clinical outcomes of this individualized approach in patients with uterine cervical cancer.

## MATERIALS AND METHODS

### Patient characteristics

We conducted a retrospective chart review of 93 consecutive patients with uterine cervical cancer who underwent treatment with radiotherapy alone or concurrent chemoradiotherapy (CCRT) with curative intent between October 2008 and May 2011. All participants provided written informed consent. The study protocol was approved by the Ethical Review Board committee of our institution. Research was conducted in accordance with the 1964 Declaration of Helsinki and its later amendments.

The inclusion criteria for the analysis were as follows: (a) histologically proven cervical cancer, (b) an International Federation of Gynecology and Obstetrics (FIGO) stage of IB1–IVA, and (c) 3D treatment planning performed for each session of brachytherapy. Of the 93 patients enrolled in this study, 13 patients (14.0%) were excluded because of treatment with palliative intent, due to the extent of local disease/distant metastases (*n* = 7), carcinomas in situ (*n* = 2), double cancer (*n* = 1), or partial 2D treatment planning of brachytherapy (*n* = 3). Thus, 80 patients (86.0%) were eligible for inclusion in the final analysis. A summary of the patients’ characteristics is provided in Table 1.

**Table 1.** Patient characteristics

Characteristic	Patients (*n* = 80)
Age at diagnosis, years (range)	59 (29–82)
Clinical stage, *n* (%)	
IB1	13 (16)
IB2	5 (6)
IIA1	6 (8)
IIA2	2 (3)
IIB	25 (31)
IIIA	1 (1)
IIIB	26 (33)
IVA	2 (3)
Tumor size at diagnosis, *n* (%)^a^	
≤4 cm (small)	29 (36)
4–6 cm (medium)	34 (43)
>6 cm (large)	17 (21)
Pelvic LNMs, *n* (%)	
Present	47 (59)
Absent	33 (41)
PALN metastases, *n* (%)	
Present	8 (10)
Absent	72 (90)
Histological type, *n* (%)	
SCC	68 (85)
ADC	11 (14)
UC	1 (1)
CCRT, *n* (%)	
Weekly cisplatin (40 mg/m^2^)	28 (35)
Weekly cisplatin (30 mg/m^2^) and paclitaxel (50 mg/m^2^)	5 (6)
None	47 (59)
Brachytherapy method, *n* (%)	
Fletcher–Suit applicator	66 (82)
Fletcher–Suit applicator with Trocar Point Needles	14 (18)

^a^ADC = adenocarcinoma, CCRT = concurrent chemoradiotherapy, LNM = lymph node metastasis, MRI = magnetic resonance imaging, PALN = paraaortic lymph node, SCC = squamous cell carcinoma, UC = undifferentiated carcinoma. Maximum tumor diameter on MRI at diagnosis.

### External beam radiotherapy

Patients were treated with combined external beam radiotherapy (EBRT) and high-dose-rate brachytherapy [3]. The clinical target volume (CTV) included the cervical tumor, uterus, parametrium, at least the upper half of the vagina, and the pelvic lymph node regions. External whole-pelvic irradiation was performed using the anteroposterior/posteroanterior field or box technique, with doses of 2 Gy per fraction, delivered five times a week. A central shielding (CS; 3 cm in width) was inserted at a total dose of 20 Gy for Stage IB1–II tumors of ≤4 cm, and 30 Gy or 40 Gy (bulky cases) for Stage II tumors of >4 cm and Stage IIIB–IVA tumors. Pelvic irradiation with CS was performed with doses of 2 Gy per fraction to a total dose of 50 Gy. For patients with gross lymph node metastases, an additional 6–10 Gy was administered to boost the external dose to the lesion to a total of 56–60 Gy. For patients with paraaortic lymph node metastases, EBRT was delivered to the paraaortic lymph node region after whole-pelvic irradiation was completed, with doses of 2 Gy per fraction to a total dose of 40 Gy, followed by a 10–16 Gy boost to the gross lymph node metastases.

### Chemotherapy

Cisplatin-based chemotherapy was administered concurrently to patients with FIGO Stage IB2–II tumors of >4 cm, FIGO Stage III–IVA tumors of any size, or pelvic lymph node metastases. The exclusion criteria for chemotherapy included an age of >75 years or severe concomitant diseases (e.g. renal dysfunction, severe diabetes, or ischemic heart disease). For the majority of eligible patients, up to 5 courses of weekly cisplatin-based chemotherapy (40 mg/m^2^) were administered concurrently with EBRT. In total, 33 patients (41.3%) received CCRT with a median of 4 (range, 3–5) courses per patient.

### Brachytherapy

**Fig. 1.**
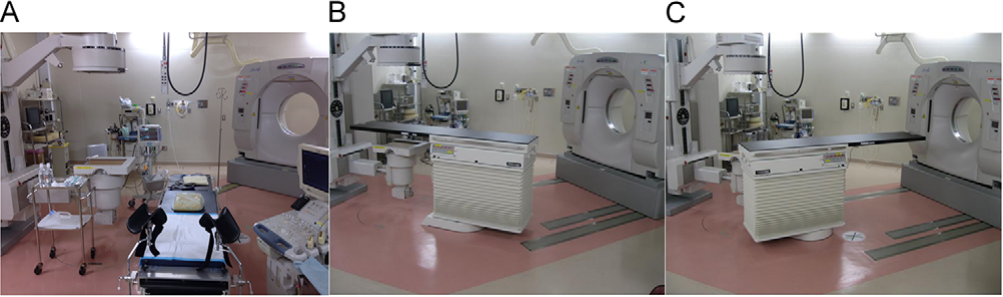
In-room computed tomography on-rail brachytherapy system showing the position of the (A) applicator insertion tool, (B) X-ray and irradiation machine, and (C) computed tomography scanner.

In addition to CS irradiation, high-dose rate brachytherapy was performed using an ^192^Ir Remote Afterloading System (microSelectron, Elekta, Stockholm, Sweden). Four fractions of brachytherapy were administered once a week, with a fraction dose of 6 Gy. In instances where the tumor response was poor, a fifth fraction of brachytherapy was considered. MRI was performed at diagnosis and within 1 week before the first brachytherapy session in each patient. The tumor response and extent of residual disease were carefully monitored through gynecological examination and were drawn on the patients’ chart before commencing brachytherapy. A Foley catheter was inserted into the bladder and inflated with 7 ml of contrast medium. A total of 100 ml of normal saline was injected immediately into the bladder before acquiring CT images and performing irradiation. After acquiring CT images, catheter clamping was delayed until treatment planning was completed. A set of Fletcher-Suit Asian Pacific applicators (tandem and half-size ovoid) was inserted under ultrasound guidance for the majority of patients. For patients with bulky and asymmetric residual tumors, Trocar Point Needles (Nucletron; Elekta, Stockholm, Sweden) were additionally inserted in combination with the tandem and ovoid applicator or uterine tandem and vaginal cylinder system [4]. Intracavitary and interstitial brachytherapy was performed in 14 patients (17.5%). Vaginal packing was used to maximize the distance from the source to the bladder and rectal walls. After implantation, the patients were placed in the supine position with their legs extended. CT on-rail images were obtained on the same couch at a 3 mm slice thickness (Fig. 1**A–C**). 

### Three-dimensional treatment planning

Three-dimensional treatment planning using CT with applicator insertion was performed for each brachytherapy session. Complete CT image datasets for brachytherapy were transferred to the Oncentra Treatment Planning System (Elekta, Stockholm, Sweden) for contouring and planning. The high-risk (HR) CTV and organs at risk (OARs) (e.g. the rectum, sigmoid colon, and bladder) were contoured according to the recommendations of The Groupe Européen de Curiethérapie and the European Society for Radiotherapy and Oncology [5, 6]. For identification of the HR CTV, findings from gynecological examinations performed at diagnosis and brachytherapy and MRI examinations performed at diagnosis and within the week before the first brachytherapy session were analyzed. As for the OARs, the outer organ contours were delineated. Delineation of the rectum included all regions from the anorectal junction to the rectosigmoid flexure.

The first plan was generated by the Oncentra Treatment Planning System, with a dose of 6 Gy per fraction normalized to Point A, based on our standard loading pattern. In instances using interstitial needles combined with intracavitary application, the dose at Point A (at the opposite side of needle placement) was normalized to 6 Gy. In principle, the number of interstitial needles used for source loading was set at one or two per unilateral tumor extension, taking the burden of needle application into consideration. A dwell position and time adaptation were initially established to optimize the first standard dose distribution and then continued with 2.5 mm stepwise additions as dwell positions within the needle. The dose distribution arising from the first standard plan was evaluated by visual inspection of the isodose lines. We predicted that a 6 Gy isodose line should cover the HR CTV in order to achieve a HR CTV D_90_ (the minimum dose delivered to 90% of the HR CTV) of >6 Gy [7, 8]. Dose adaptation was initially based on dose changes at Point A. If dose adaptation to Point A could not be achieved as intended, manual optimization of dwell positions and dwell weights in the tandem and ovoid applicator and needles was performed to improve dosimetry. DVH parameters were calculated with respect to the HR CTV D_90_ and OARs D_2cm^3^_ (the minimum dose delivered to the highest irradiated 2 cm^3^ area), as per the recommendations of The Groupe Européen de Curiethérapie and the European Society for Radiotherapy and Oncology [5, 6].

### Dose–volume histogram parameters

The cumulative doses of EBRT and brachytherapy were summarized and normalized to a biological equivalent dose of 2 Gy per fraction (EQD2) using a linear–quadratic model with an alpha/beta of 3 Gy for the OARs and 10 Gy for the tumors. In this study, 3D DVH parameters of the HR CTV and OARs were calculated by adding the biologically equivalent doses of whole-pelvic EBRT and all of the brachytherapy sessions. Pelvic irradiation doses after CS were not included because the central core of the cervical tumor and adjacent rectum and bladder received much fewer doses with gradient after CS. DVH parameters on dose constraints for the OARs were not determined since, to date, there has been no definitive evidence regarding dose constraints for the rectum and bladder in the EBRT regimen with CS. Instead, from our initial clinical experiences, we aimed to achieve cumulative doses of EBRT and brachytherapy of >60 Gy (EQD2) for HR CTV D_90_, <75 Gy (EQD2) for D_2 cm^3^_ of the rectum, and <90 Gy (EQD2) for D_2 cm^3^_ of the bladder.

### Follow-up

Patients were followed-up every 1–3 months for the initial 2 years and every 3–6 months for the subsequent 3 years. Disease status and the extent of late toxicities were assessed at each follow-up examination by taking the patient's history, conducting a physical examination, and/or performing appropriate laboratory and radiological tests. Suspected recurrent cervical tumors were confirmed by biopsy wherever possible. Late toxicities were classified according to the National Cancer Institute Common Terminology Criteria for Adverse Events, version 3.0 [9].

### Statistical analyses

Local control (LC) was measured from the date of commencing of therapy to the date of the first local recurrence or last follow-up. Pelvic disease progression was defined as follows: (a) pelvic recurrence after assessment of complete response; (b) pelvic disease progression with an increase of >20% in the size of the target lesions, as assessed by MRI; or (c) initiation of salvage treatment for pelvic disease, irrespective of pathological findings. Pelvic progression-free survival (PFS) was measured from the date of commencing therapy to the date of the first pelvic disease progression, including local recurrence, or last follow-up. Overall survival (OS) was measured from the date of commencing therapy to the date of death from any cause or last follow-up. LC, pelvic PFS, and OS rates were calculated using the Kaplan–Meier method. Differences in the survival curves were evaluated by the log-rank test, and differences in the DVH parameters were assessed using an analysis of variance test. A *P* < 0.05 was considered statistically significant. All statistical analyses were conducted using Statistical Package for the Social Sciences for Mac, software version 16.0 (SPSS Inc., Chicago, IL, USA).

## RESULTS

### Local control, pelvic progression-free survival, and overall survival rates

**Fig. 2.**
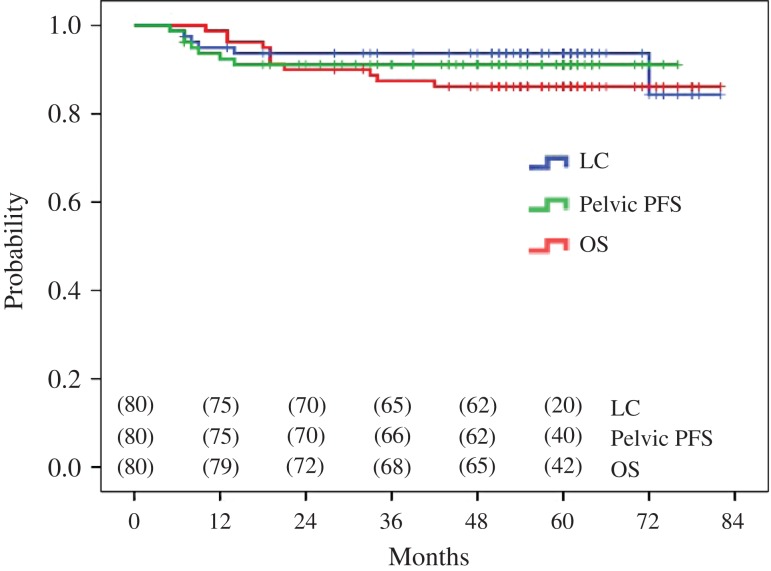
Five-year local control (LC; blue line), pelvic progression-free survival (PFS; green line), and overall survival (OS; red line) rates of all (*n* = 80) uterine cervical cancer patients combined.

**Fig. 3.**
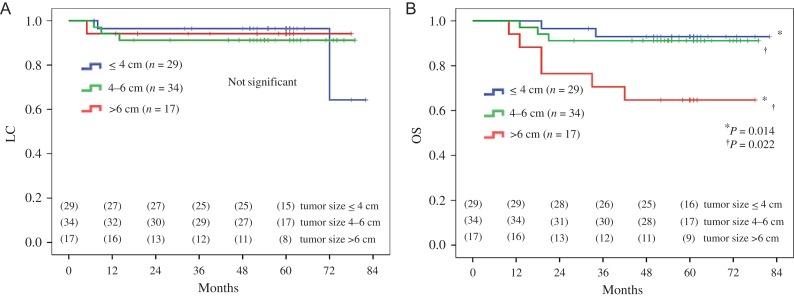
Five-year (A) local control (LC) and (B) overall survival (OS) rates of uterine cervical cancer patients stratified according to tumor size (≤4 cm [*n* = 29], blue line; 4–6 cm [*n* = 34], green line; and > 6 cm [*n* = 17], red line).

**Fig. 4.**
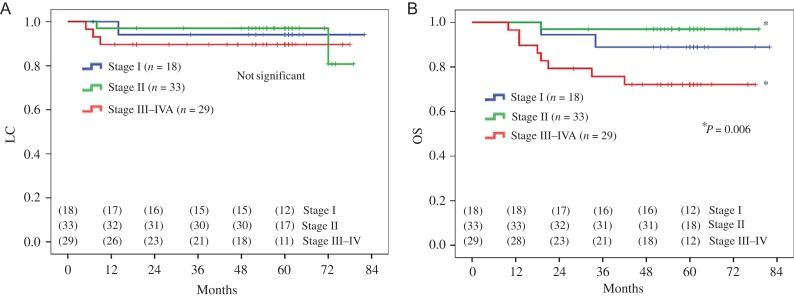
Five-year (A) local control (LC) and (B) overall survival (OS) rates of uterine cervical cancer patients stratified according to clinical stage [Stage I (*n* = 18), blue line; Stage II (*n* = 33), green line; and Stage III–IVA (*n* = 29), red line].

The median follow-up durations were 60 (range, 44–82) months and 60 (range, 10–82) months for surviving patients only and all patients combined, respectively. Six patients (7.5%) developed local recurrences (squamous cell carcinoma [*n* = 5 patients] and adenocarcinoma [*n* = 1 patient]). Of these, 2 patients (33.3%) were treated with salvage interstitial brachytherapy. Both were alive without disease progression at the time of analysis. Three patients (3.8%) developed pelvic lymph node recurrences, and 14 patients (17.5%) developed distant metastases. None of the patients developed both local and pelvic lymph node recurrences. Eleven patients (13.8%) were deceased at the last follow-up. Nine patients (81.8%) died of cervical cancer, and the remaining 2 patients (18.2%) died of other malignancies). The 5-year LC, pelvic PFS, and OS rates of all patients combined were 94%, 90% and 86%, respectively (Fig. 2). The 5-year LC, pelvic PFS, and OS rates according to tumor size were 96%, 93% and 93%; 91%, 91% and 91%; and 94%, 82% and 65% for tumors ≤4 cm (*n* = 29 patients); tumors 4–6 cm (*n* = 34 patients); and tumors >6 cm (*n* = 17 patients), respectively (Fig. 3**A–B and**Supplementary Fig. 1**A**). The 5-year LC, pelvic PFS and OS rates according to FIGO stage were 94%, 94% and 89%; 97%, 94% and 97%; and 90%, 83% and 72% for Stage I (*n* = 18 patients); Stage II (*n* = 33 patients); and Stage III–IVA (*n* = 29 patients), respectively (Fig. 4**A–B and**Supplementary Fig. 1**B**). 

#### Late toxicities

Three patients (3.8%) developed Grade 2 rectal toxicity [D_2cm^3^_ of the rectum, 70 Gy, 70 Gy and 59 Gy (EQD2), respectively). Three patients (3.8%) developed Grade 2 urinary toxicity (D_2cm^3^_ of the bladder, 84 Gy, 74 Gy and 73 Gy [EQD2], respectively). One patient (1.3%) with liver cirrhosis developed Grade 3 urinary toxicity [D_2cm^3^_ of the bladder, 96 Gy (EQD2)] that required blood transfusion and hyperbaric oxygen therapy. No other late toxicities of Grade 2 or higher were reported in the rectum or bladder.

### Dose–volume histogram parameters

The actual DVH parameters are summarized in Table 2. The proportions of patients with a HR CTV D_90_ of >60 Gy (EQD2), D_2cm^3^_ of the rectum of <75 Gy (EQD2), and D_2cm^3^_ of the bladder of <90 Gy (EQD2) were 90%, 99% and 93%, respectively.

**Table 2.** Actual dose–volume histogram parameters

Parameter	Mean dose ± SD (Gy)^a^		*P*-value
HR CTV (D_90_)			
≤4 cm (small)	69.0 ± 11.9	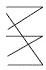	0.958
4–6 cm (medium)	68.4 ± 5.5		0.723
>6 cm (large)	66.5 ± 6.4		0.593
Rectal dose (D_2cm^3^_)			
≤4 cm (small)	48.4 ± 11.3	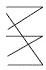	0.002^*^
4–6 cm (medium)	57.1 ± 9.5		0.098
>6 cm (large)	63.2 ± 7.4		<0.001^*^
Bladder dose (D_2cm^3^_)			
≤4 cm (small)	67.9 ± 12.9	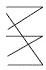	0.081
4–6 cm (medium)	73.9 ± 9.1		0.123
>6 cm (large)	80.2 ± 9.7		0.001^*^

^a^CTV = clinical target volume, D_2cm3_ = the minimum dose delivered to the highest irradiated 2 cm^3^ volume, D_90_ = the minimum dose delivered to 90% of the HR CTV, HR = high risk, OARs = organs at risk, SD = standard deviation. The total doses of external body radiotherapy and brachytherapy were calculated and normalized to a biological equivalent dose of 2 Gy per fraction using a linear quadratic model with an alpha/beta of 3 Gy for the OARs and an alpha/beta of 10 Gy for the HR CTV. The doses of pelvic irradiation with central shielding were not included.

## DISCUSSION

Brachytherapy remains essential in the definitive treatment of cervical cancer, although there are regional- and community-specific variations in its application (e.g. dose rates, fractionation schedules, and applicator type [10]). In spite of the variations potentially derived from tradition in brachytherapy, excellent LC rates (89–98%) and minimal late toxicities have been reported [11–25], irrespective of the imaging modality used (i.e. CT or MRI) in treatment planning for 3D IGBT (Table 3). In particular, as noted in the present study, the long-term effectiveness of LC has recently been established [23–25].

**Table 3:** Review of recent clinical outcomes of 3D image-guided brachytherapy for uterine cervical cancer

Author (reference)	Year	Number of patients	Imaging for 3D planning	Median follow-up (months)	LC rate	Late toxicity Grade 3 or higher
Tan *et al*. [11]	2009	28	CT	23	96%	11%
Kang *et al*. [12]	2010	97	CT	41	97% (3-year)	4%
Pötter *et al*. [13]	2011	156	MRI	42	95% (3-year)	4% (rectum), 2% (bladder)
Charra-Brunaud *et al*. [14]	2012	117	CT (82%), MRI (18%)	24	79% (2-year)	3%
Tharavichitkul *et al*. [15]	2013	47	CT (68%), MRI (32%)	26	98%	2%
Lindegaard *et al*. [16]	2013	140	MRI	36	91% (3-year)	3% (GI tract), 1% (urinary tract)
Dyk *et al*. [17]	2014	134	MRI	29	82%	NR
Murakami *et al*. [18]	2014	51	CT	39	92% (3-year)	2% (GI tract), 0% (bladder)
Rijkmans *et al*. [19]	2014	83	CT (52%), MRI (48%)	42	NR	8%
Gill *et al*. [20]	2015	128	MRI	24	92% (2-year)	1%
Simpson *et al*. [21]	2015	76	CT fused with MRI	17	95%	2%
Mazeron *et al*. [22]	2015	225	CT (10%), MRI (90%)	39	87%	NR
Zolciak-Siwinska *et al*. [23]	2016	216	CT	52	90% (5-year)	4% (rectum), 3% (bladder)
Ribeiro *et al*. [24]	2016	170	CT (4%), MRI (96%)	37	96% (5-year)	7% (rectosigmoid), 6% (urinary tract)
Sturdza *et al*. [25]	2016	731	CT (19%), MRI (81%)	43	89% (5-year)	7% (GI tract), 5% (bladder)
Present study	2016	80	CT	60	94% (5-year)	0% (rectosigmoid), 1% (bladder)

CT = computed tomography, GI = gastrointestinal, LC = local control, MRI = magnetic resonance imaging, NR = not reported.

Previous studies [11, 12, 14, 16, 19, 26] have demonstrated that 3D IGBT significantly improves the LC rates of locally advanced cervical cancer patients as compared with historical controls. In a prospective Japanese Gynecologic Oncology Group 1066 (JGOG1066) study [27] of CCRT, using the Japanese standard regimen for locally advanced cervical cancer, a fixed dose of 6 Gy per fraction for 4 fractions was prescribed to Point A in 2D treatment planning of brachytherapy. The 2-year pelvic PFS rates for tumors <5 cm, 5–7 cm and >7 cm were 77%, 69% and 39%, respectively. LC rates according to tumor size were not presented in the JGOG1066 study [27]. However, considering lower LC rates are associated with a poorer pelvic PFS rate, the JGOG1066 study [27] indicated that a fixed dose of 6 Gy to Point A was ineffective for larger tumors. Conversely, in the present study, the 5-year pelvic PFS rates for tumors ≤4 cm, 4–6 cm and >6 cm were 93%, 91% and 82%, respectively. Comparable EBRT schedules were used in the present study as in the JGOG1066 study [27], with or without chemotherapy. However, in the 3D treatment planning of brachytherapy, we predicted that a 6 Gy isodose line should cover the HR CTV on a visual dose distribution curve and that the HR CTV D_90_ would be >6 Gy. Consequently, there were no significant differences in the HR CTV D_90_ among patients with different sized tumors. In the present study, we demonstrated a relatively higher pelvic PFS rate compared with that of the JGOG1066 study [27], irrespective of tumor size, by adopting 3D IGBT.

In the present study, an advanced FIGO stage was associated with a significantly poorer OS rate (89%, 97% and 72% for Stage I, Stage II and Stage III patients, respectively). In contrast, recent studies [23, 25] using 3D IGBT revealed 5-year OS rates of 83%, 70–78% and 42–52% for Stage I, Stage II and Stage III patients, respectively. A direct comparison between our study and previously published studies [23, 25] on the use of 3D IGBT is not possible due to potential underlying biases (e.g. patient selection, tumor evaluation, the use of combined systemic therapy, and the biological nature of the disease). Although the LC rates are comparable among these studies, the relatively higher OS rates in the present study will need to be explained in future studies.

To date, definitive evidence is lacking regarding the dose constraints of the rectum and bladder for 3D IGBT using the Japanese treatment regimen, mainly owing to the low incidence rates of severe late toxicities [28, 29]. In a Japanese study [18], the incidence rates of late toxicities of Grade 3 or higher in the rectosigmoid and bladder were ≤2% and ≤1%, respectively. In a recent observational study by the Leuven Cancer Institute [24] and a retrospective international study (RetroEMBRACE) [25], the incidence rates of late toxicities of Grade 3 or higher in the rectosigmoid and bladder were 7% and 5–6%, respectively (Table 3). The D_2cm^3^_ of the rectum and bladder in these studies [18, 24, 25] was 61–64 Gy (EQD2) and 81–83 Gy (EQD2), respectively, which appear to be higher than the doses recorded in our study. In a phantom study [30] evaluating the composite dose and DVH parameters for brachytherapy and EBRT, the contribution of CS doses to the HR CTV D_90_, bladder D_2cm^3^_, and rectal D_2cm^3^_ was 24–56%, 28–32% and 9%, respectively, in instances of 3 cm CS for various sizes of the HR CTV. Our present study suggests that the use of CS may be advantageous in achieving a higher dose ratio, especially with respect to the HR CTV D_90_ and rectal D_2cm^3^_ in certain clinical settings. However, there are many uncertainties in the clinical practice of radiotherapy and CCRT for cervical cancer, depending on the tumor size, tumor topography, and rectal location. Therefore, further analysis of DVH parameters, including the contribution of CS doses to the HR CTV and OARs, is required.

In comparing recent clinical trial data [11–13, 16–18, 20, 23] of CT-based and MRI-based 3D IGBT, the LC rate and incidence rate of severe late toxicities appeared to be comparable (Table 3). Evidently, CT alone is inferior to MRI in visualizing cervical tumors. A comparison of CT and MRI for CTV delineation in 3D IGBT for cervical cancer has revealed that CT-based contouring overestimates the contour width [31]. However, such overestimations may be improved by including information from 3D documentation of physical examinations and diagnostic MRI without applicator insertion before brachytherapy [32, 33]. Moreover, a combination of MRI-based 3D IGBT for the first session and CT-based 3D IGBT for the subsequent sessions has yielded excellent LC and OS rates [34]. In the present study, CT-based 3D IGBT was performed, which was supported by gynecological examinations, ultrasound guidance, and MRI at diagnosis and within 1 week before the first session of brachytherapy. We postulate that precise tumor evaluation through multiple diagnostic approaches and the adaptive use of interstitial needles will contribute to higher LC rates and/or reductions in severe late toxicities, irrespective of tumor size and topography [4, 8].

In the RetroEMBRACE study [25], the mean HR CTV D90 values in different centers ranged from 71 to 95 Gy (EQD2), which were relatively high compared with the results of our study. There are several possible explanations for the comparable LC rates despite a lower HR CTV D90 in our study. First, the HR CTV D90 is likely to have been underestimated due to the use of CS in EBRT (e.g. unshielded lateral extension of the tumor irradiated with 50 Gy of EBRT), as demonstrated in a phantom study [30]. Second, delivering a high dose by placing the brachytherapy source in the target tumor may have contributed to local control, even after commencing CS. We think that DVH parameters other than the HR CTV D90 should be evaluated. Third, the method of contouring the HR CTV D90 differs between CT-and MRI-acquired images. However, as discussed previously, the risk of overestimating the HR CTV D90 was minimized in our study by using repeated diagnostic MRI and gynecological examinations.

To the best of our knowledge, we are the first to report on the long-term clinical outcomes of 3D IGBT using an in-room CT-based system for cervical cancer. In our approach, patient transfer is not necessary between the acquisition of CT images and applicator insertion or irradiation. This system is clinically advantageous in reducing the number of applicator displacements, the burden for the patient and medical staff, and the time delay for irradiation.

In conclusion, 3D IGBT using an in-room CT on-rail brachytherapy system produced excellent LC rates (irrespective of tumor size and clinical stage) in cervical cancer patients, without an increase in the incidence of severe late toxicities. Prospective evaluation in a multicenter setting will be required to confirm these findings.

## SUPPLEMENTARY DATA

Supplementary data are available at the *Journal of Radiation Research* online.

## FUNDING

This work was supported by grants-in-aid from the Ministry of Education, Culture, Sports, Science, and Technology of Japan for Scientific Research in Innovative Areas and the Japan Society for the Promotion of Science for Young Scientists [KAKENHI; grant number 26461879 to T.O.].

## CONFLICT OF INTEREST

None.

## Supplementary Material

Supplementary Data
