# Supplementary material for: In-room computed tomography–based brachytherapy for uterine cervical cancer: results of a 5-year retrospective study
Source: J Radiat Res. 2016 Dec 16;58(4):543–51. doi: 10.1093/jrr/rrw121 (PMC5766167; doi:10.1093/jrr/rrw121)

Supplementary data

Figure 1: Pelvic progression-free survival according to tumor size or clinical stage.

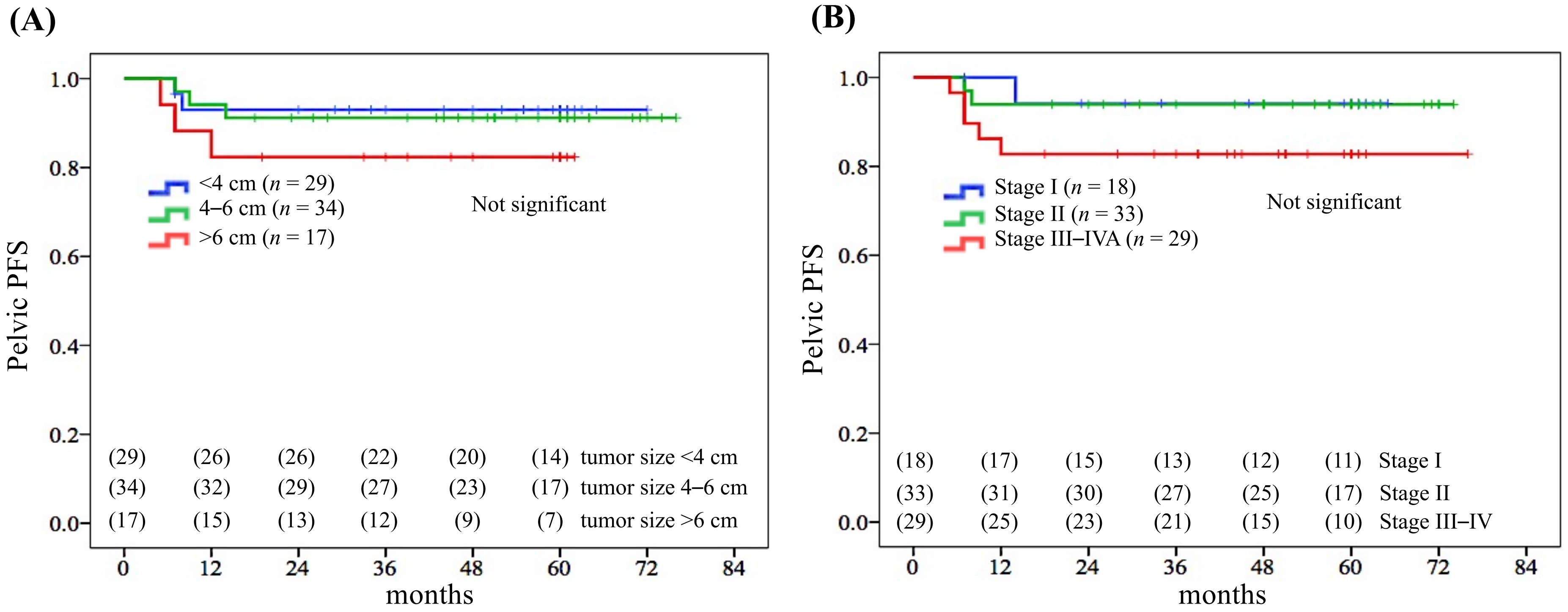

Supplement: Supplementary Data [file jrr_suppl_data.pdf]
